# Supplementary material for: Meta-Analysis and Gene Set Enrichment Relative to ER Status Reveal Elevated Activity of MYC and E2F in the “Basal” Breast Cancer Subgroup
Source: PLoS One. 2009 Mar 9;4(3):e4710. doi: 10.1371/journal.pone.0004710 (PMC2650420; doi:10.1371/journal.pone.0004710)
Supplement: Dataset S1 — GSEA results from first screen. The file contains a zipped archive of the HTML results of the second GSEA screen. Reports for enriched or depleted datasets may be accessed from the “index.html” link within the my_analysis.GseaPreranked.1219975950625.rpt.zip ZIP archive. Enrichment plots are available for each of the 50 most enriched and depleted gene sets. (13.32 MB ZIP) [file pone.0004710.s001.zip › my_analysis.GseaPreranked.1219975950625.rpt.zip!//P21_P53_ANY_DN.html]

Details for gene set P21\_P53\_ANY\_DN[GSEA]

|  || Dataset | zSco.ERneg.to.ERpos\_collapsed |
| Phenotype | NoPhenotypeAvailable |
| Upregulated in class | na\_pos |
| GeneSet | P21\_P53\_ANY\_DN |
| Enrichment Score (ES) | 0.7568868 |
| Normalized Enrichment Score (NES) | 2.7293515 |
| Nominal p-value | 0.0 |
| FDR q-value | 0.0 |
| FWER p-Value | 0.0 |
Table: GSEA Results Summary

  

Fig 1: Enrichment plot: P21\_P53\_ANY\_DN      
 Profile of the Running ES Score & Positions of GeneSet Members on the Rank Ordered List

  

| PROBE | GENE SYMBOL | GENE\_TITLE | RANK IN GENE LIST | RANK METRIC SCORE | RUNNING ES | CORE ENRICHMENT || 1 | TTK |  |  | 11 | 7.059 | 0.0443 | Yes |
| 2 | NCAPG |  |  | 29 | 6.628 | 0.0853 | Yes |
| 3 | BUB1 |  |  | 41 | 6.445 | 0.1257 | Yes |
| 4 | AURKB |  |  | 76 | 6.038 | 0.1617 | Yes |
| 5 | MKI67 |  |  | 98 | 5.871 | 0.1977 | Yes |
| 6 | MCM4 |  |  | 180 | 5.436 | 0.2263 | Yes |
| 7 | MCM6 |  |  | 198 | 5.317 | 0.2590 | Yes |
| 8 | EXO1 |  |  | 218 | 5.239 | 0.2910 | Yes |
| 9 | TPX2 |  |  | 231 | 5.180 | 0.3232 | Yes |
| 10 | CEP55 |  |  | 279 | 4.995 | 0.3516 | Yes |
| 11 | MCM7 |  |  | 289 | 4.970 | 0.3827 | Yes |
| 12 | CDC25B |  |  | 340 | 4.826 | 0.4098 | Yes |
| 13 | ASPM |  |  | 359 | 4.767 | 0.4389 | Yes |
| 14 | AURKA |  |  | 460 | 4.483 | 0.4600 | Yes |
| 15 | DLG7 |  |  | 521 | 4.306 | 0.4830 | Yes |
| 16 | PRC1 |  |  | 543 | 4.253 | 0.5086 | Yes |
| 17 | MCM2 |  |  | 630 | 4.080 | 0.5282 | Yes |
| 18 | SMC2 |  |  | 651 | 4.028 | 0.5524 | Yes |
| 19 | MCM3 |  |  | 656 | 4.019 | 0.5778 | Yes |
| 20 | NCAPD3 |  |  | 661 | 4.016 | 0.6031 | Yes |
| 21 | CCDC99 |  |  | 737 | 3.863 | 0.6222 | Yes |
| 22 | TYMS |  |  | 829 | 3.738 | 0.6392 | Yes |
| 23 | FAT2 |  |  | 981 | 3.509 | 0.6503 | Yes |
| 24 | UBE2C |  |  | 994 | 3.489 | 0.6716 | Yes |
| 25 | TUBB3 |  |  | 1109 | 3.350 | 0.6845 | Yes |
| 26 | CDC2 |  |  | 1259 | 3.182 | 0.6936 | Yes |
| 27 | ZBTB5 |  |  | 1442 | 3.006 | 0.6991 | Yes |
| 28 | ATAD2 |  |  | 1532 | 2.917 | 0.7110 | Yes |
| 29 | WDHD1 |  |  | 1576 | 2.872 | 0.7261 | Yes |
| 30 | KIAA1794 |  |  | 1724 | 2.747 | 0.7326 | Yes |
| 31 | C14ORF106 |  |  | 1844 | 2.651 | 0.7406 | Yes |
| 32 | PBK |  |  | 1853 | 2.647 | 0.7569 | Yes |
| 33 | TCN2 |  |  | 3279 | 1.803 | 0.6611 | No |
| 34 | SERPINI2 |  |  | 3363 | 1.765 | 0.6661 | No |
| 35 | KNTC1 |  |  | 3562 | 1.679 | 0.6619 | No |
| 36 | HMGB2 |  |  | 3883 | 1.558 | 0.6477 | No |
| 37 | VEGF |  |  | 3911 | 1.549 | 0.6556 | No |
| 38 | RACGAP1 |  |  | 4097 | 1.475 | 0.6511 | No |
| 39 | NPM1 |  |  | 4288 | 1.397 | 0.6457 | No |
| 40 | TUBB4 |  |  | 5352 | 1.025 | 0.5722 | No |
| 41 | BIRC2 |  |  | 5640 | 0.933 | 0.5565 | No |
| 42 | PARP2 |  |  | 7734 | 0.219 | 0.4002 | No |
| 43 | TUBA3 |  |  | 8675 | -0.160 | 0.3305 | No |
| 44 | ZNF84 |  |  | 9244 | -0.429 | 0.2904 | No |
| 45 | HNRPA2B1 |  |  | 9531 | -0.578 | 0.2725 | No |
| 46 | CYR61 |  |  | 10171 | -0.940 | 0.2304 | No |
| 47 | TRIM44 |  |  | 10338 | -1.054 | 0.2246 | No |
Table: GSEA details [plain text format]

  

Fig 2: P21\_P53\_ANY\_DN: Random ES distribution      
 Gene set null distribution of ES for **P21\_P53\_ANY\_DN**
